# Kawasaki shock syndrome: a case report

**DOI:** 10.1186/1546-0096-12-S1-P350

**Published:** 2014-09-17

**Authors:** Edoardo Marrani, Teresa Giani, Valeria Paganelli, Gabriele Simonini, Ilaria Pagnini, Giovanni Calabri, Rolando Cimaz

**Affiliations:** 1 Pediatric Rheumatology, Anna Meyer Children Hospital, Firenze, Italy; 2 Pediatric Cardiology, Anna Meyer Children Hospital, Firenze, Italy

## Introduction

Kawasaki disease is the second most common systemic vasculitis of childhood and may result in life-threatening coronary artery abnormalities in up to 25% of untreated patients. Adolescents often present with an atypical/incomplete presentation of KD with a delayed diagnosis. Kawasaki Shock syndrome (KDSS) is a rare KD presentation and has been recently defined as the presence of any of the following conditions: systolic hypotension (< -2 SD blood pressure defined for age and sex), a decrease in systolic blood pressure from baseline of >20% or clinical signs of poor perfusion with accompanying features of KD [1].

## Objectives

To describe a case of KDSS and to increase awareness for this rare condition among paediatric rheumatologist and intensivists.

## Methods

We report the case of 15-year-old boy presenting with atypical KD complicated by severe shock syndrome and successfully treated with high-dose intravenous immunoglobulins (IVIG).

## Results

A 15-year-old boy presented to another hospital after four days of high-grade fever, diffuse abdominal pain, diarrhoea and a maculo-papular cutaneous rash. He received antibiotic therapy and underwent laparoscopic abdominal exploration in the suspicion of acute appendicitis, which was ruled out. The postoperative period was complicated by fever persistence and a by general clinical worsening with clinical signs of shock and of left ventricular dysfunction; a presumptive diagnosis of viral myocarditis was made and the boy was transferred to our ICU where he required mechanical ventilation, fluid resuscitation and inotropic support. Microbiological test failed to identify any infectious agent. Because of the persistence of high grade fever, the sequential appearance of other four KD criteria (cheilitis, rash, non-secretive conjunctivitis, oedema of hands and feet) and the demonstration of left coronary artery hyperechogenicity, a diagnosis of KDSS was made and he was administered two courses of IVIG with rapid improvement. Eventually he was discharged with low-dose aspirin; two weeks later he showed periungual peeling of fingers and toes. No further complications appeared, and at last follow up visit, 24 months after diagnosis, he was completely asymptomatic with normal laboratory tests and echo-cardiogram.

## Conclusion

KDSS is a rare aetiology for shock in childhood; the diagnosis could be missed because of its atypical presentation. Because of the need for proper diagnosis and rapid treatment, paediatricians and paediatric intensivists should maintain a high index of suspicion.

## Disclosure of interest

None declared
